# ATR-16 syndrome: mechanisms linking monosomy to phenotype

**DOI:** 10.1136/jmedgenet-2019-106528

**Published:** 2020-01-31

**Authors:** Christian Babbs, Jill Brown, Sharon W Horsley, Joanne Slater, Evie Maifoshie, Shiwangini Kumar, Paul Ooijevaar, Marjolein Kriek, Amanda Dixon-McIver, Cornelis L Harteveld, Jan Traeger-Synodinos, Andrew O M Wilkie, Douglas R Higgs, Veronica J Buckle

**Affiliations:** 1 MRC Molecular Haematology Unit, MRC Weatherall Institute of Molecular Medicine, University of Oxford, Oxford, UK; 2 IGENZ Ltd, Auckland, New Zealand; 3 Department of Clinical Genetics, Leiden University Medical Center, Leiden, The Netherlands; 4 Department of Medical Genetics, National and Kapodistrian University of Athens, Athens, Greece; 5 Clinical Genetics Group, MRC Weatherall Institute of Molecular Medicine, University of Oxford, Oxford, UK; 6 Craniofacial Unit, Oxford University Hospitals NHS Trust, John Radcliffe Hospital, Oxford, UK

**Keywords:** ATR16, thalassemia, CNV, developmental delay

## Abstract

**Background:**

Deletions removing 100s–1000s kb of DNA, and variable numbers of poorly characterised genes, are often found in patients with a wide range of developmental abnormalities. In such cases, understanding the contribution of the deletion to an individual’s clinical phenotype is challenging.

**Methods:**

Here, as an example of this common phenomenon, we analysed 41 patients with simple deletions of ~177 to ~2000 kb affecting one allele of the well-characterised, gene dense, distal region of chromosome 16 (16p13.3), referred to as ATR-16 syndrome. We characterised deletion extents and screened for genetic background effects, telomere position effect and compensatory upregulation of hemizygous genes.

**Results:**

We find the risk of developmental and neurological abnormalities arises from much smaller distal chromosome 16 deletions (~400 kb) than previously reported. Beyond this, the severity of ATR-16 syndrome increases with deletion size, but there is no evidence that critical regions determine the developmental abnormalities associated with this disorder. Surprisingly, we find no evidence of telomere position effect or compensatory upregulation of hemizygous genes; however, genetic background effects substantially modify phenotypic abnormalities.

**Conclusions:**

Using ATR-16 as a general model of disorders caused by CNVs, we show the degree to which individuals with contiguous gene syndromes are affected is not simply related to the number of genes deleted but depends on their genetic background. We also show there is no critical region defining the degree of phenotypic abnormalities in ATR-16 syndrome and this has important implications for genetic counselling.

## Introduction

Cytogenetic, molecular genetic and more recently, next-generation sequencing (NGS) approaches have revealed CNVs in the human genome ranging from 1 to 1000s of kilobases (kb).^1 2^ CNVs are common in normal individuals and have been identified in ~35% of the human genome.^1^ When present as hemizygous events, in phenotypically ‘normal’ individuals, these imbalances are considered benign; however, CNVs are also among the most common causes of human genetic disease and they have been associated with a wide range of developmental disabilities present in up to 14% of the population.^3^

CNVs have been shown to play an important role in neurodevelopmental disorders including autism spectrum disorder, bipolar disorder and schizophrenia as well as influencing broader manifestations such as learning disabilities, abnormal physical characteristics and seizures.^4^ Some CNVs occur recurrently in association with one particular phenotype: for example, deletions within 16p11.2 and/or chromosome 22q are frequently associated with autism, and deletions within 15q13.3 and 1q21.1 are found in schizophrenia. However, the impact of most CNVs on phenotype is much less clear.^4^ Difficulty in interpreting CNVs particularly occurs when they result from complex rearrangements such as those associated with unbalanced translocations, inversions and imprinting effects.

To understand the principles and mechanisms by which CNVs lead to developmental abnormalities we have simplified the issue by studying the relationship between uncomplicated deletions within the region ~0.3 to ~2 Mb in the subtelomeric region of chromosome 16 and the resulting phenotypes. The 41 individuals studied here (comprising 12 new and 29 previously reported cases) represent a cohort of patients with the α-thalassaemia mental retardation (MR) contiguous gene syndrome, involving the chromosomal region 16p13.3, termed ATR-16 syndrome (MIM 141750).^5^

Individuals studied here have monosomy for various extents of the gene-rich distal region at 16p13.3 and all individuals with ATR-16 syndrome have α-thalassaemia because two of the four paralogous α-globin genes are deleted (--/αα) and this manifests as mild hypochromic microcytic anaemia. In combination with a common small deletion involving one α-gene on the non-paralogous allele (--/-α), patients may have a more severe form of α-thlassaemia referred to as HbH disease.^6^ Some patients also have MR, developmental abnormalities and/or speech delay and facial dysmorphism. The most severe cases also manifest abnormalities of the axial skeleton. By precisely defining the 16p13.3 deletions in 11 cases (with a further 8 characterised by microarray) we address whether the associated neurological and developmental defects are simply related to the size of the deletions and the number of genes removed and whether there are critical haploinsufficient genes within this region. Our findings suggest that while the loss of an increased number of genes tends to underlie more severe phenotypic abnormalities, the genetic background in which these deletions occur contributes to the occurrence of MR and developmental abnormalities.

Finally, this subgroup of ATR-16 patients also allowed us to address two long-standing questions associated with large subcytogenetic deletions: those of compensatory gene expression and telomere proximity effect (TPE) in cases of telomere repaired chromosomal breakages.

## Methods

### Patients

Here, we focus on a cohort of patients with pure monosomy within 16p13.3 to clarify the effect of the deletion. In this work, we identify or refine the breakpoints of 14 deletions in a total of 19 individuals (including 9 cases from 4 families designated TN, TY, CS and SH and 10 singleton cases (OY, LA, YA, BA, NL, CJ, MY, BAR, IM and LIN)). In addition, we review 11 cases from 2 families (designated BF and F) and 11 singleton cases (JT, CV, AB, GZ, GIB, DO, SCH, PV, FT, BO, HN). Together this amounts to 20 familial cases from 5 pedigrees and 21 singletons amounting to a total of 41 patients.

### Fluorescent in situ hybridisation

FISH studies were carried out on fixed chromosome preparations as previously described^7^ from each patient using a series of cosmids covering the terminal 2 Mb of chromosome 16p (online supplementary table 1). FISH studies were also performed using probes specific for the subtelomeric regions of each chromosome in order to exclude any cryptic chromosomal rearrangements. Subtelomeric rearrangements were detected as previously described.^8^ ^9^

10.1136/jmedgenet-2019-106528.supp1 Supplementary data

### Southern blotting

Single copy probes labelled with α^32^P-dCTP were synthesised and used to hybridise Southern blots of DNA isolated from transformed lymphoblastoid cells.

### PCR detection of chromosomal deletions

DNA was extracted from mouse/human hybrid cell lines or transformed lymphoblastoid lines. Based on FISH results with chromosome 16 cosmids, primer pairs were designed located at regular intervals across the breakpoint clone. To refine the 16p breakpoint, PCR amplification was performed using normal and abnormal patient hybrid DNA obtained from mouse erythroleukaemia (MEL cells) fused to patient cells and selected to contain a single copy of human chromosome 16 generated as previously described^10^ as template. A positive PCR indicated the sequence was present; a negative PCR indicated it was deleted.

### Telomere-anchored PCR amplification

Telomere-anchored PCR was undertaken using a primer containing canonical telomeric repeats in conjunction with a reverse primer specific for the normal 16p sequence (primer sequences provided in online supplementary table 2). Telomere repeat primers hybridise at any location in telomere repeats so heterogeneous amplification products are produced. Amplification products were purified and digested with restriction endonucleases BamHI or EcoRI and products ligated into appropriately prepared pBluescript. Resulting colonies were screened for inserts and DNA Sanger sequenced.

### Quantification of gene expression

Total RNA was isolated from Epstein-Barr virus transformed lymphoblastoid cell lines for 11 patients (OY, TY, BA, MY, BO, CJ, YA, TN (Pa), SH (Pa), LIN and IM) and 20 control individuals using TRI reagent. In the case of OY, the genes *MRPL28*, *TMEM8*, *NME4*, *DECR2* and *RAB11FIP3* were excluded from the analysis as they are proximal to the deleted region and are not hemizygous in this patient. In the case of CJ, *POLR3K*, *C16ORF33*, *MPG* and *C16ORF35* are excluded as they are distal to the interstitial deletion in this patient. cDNA synthesis was performed with the AffinityScript kit (Stratagene). Where gene expression was measured by quantitative real-time PCR, TaqMan Gene Expression Assays Applied Biosystems (ABI, www.appliedbiosystems.com) were used. Genes and assay numbers are given in online supplementary information.

### Microarray analysis

Details of the microarray platforms used for each patient are given in online supplementary information.

### Whole genome sequencing

Whole genome sequencing (WGS) was carried out, using DNA from the three affected members of the TN family and YA, at Edinburgh Genomics, The University of Edinburgh. The pathogenicity of each variant was given a custom deleterious score based on a six-point scale,^11^ calculated using output from ANNOVAR.^12^ This was used to prioritise variants present in the hemizygous region of chr16p13.3 in each patient and also genome-wide.

## Results

### Clinical features of ATR-16 syndrome

**Figure 1:**
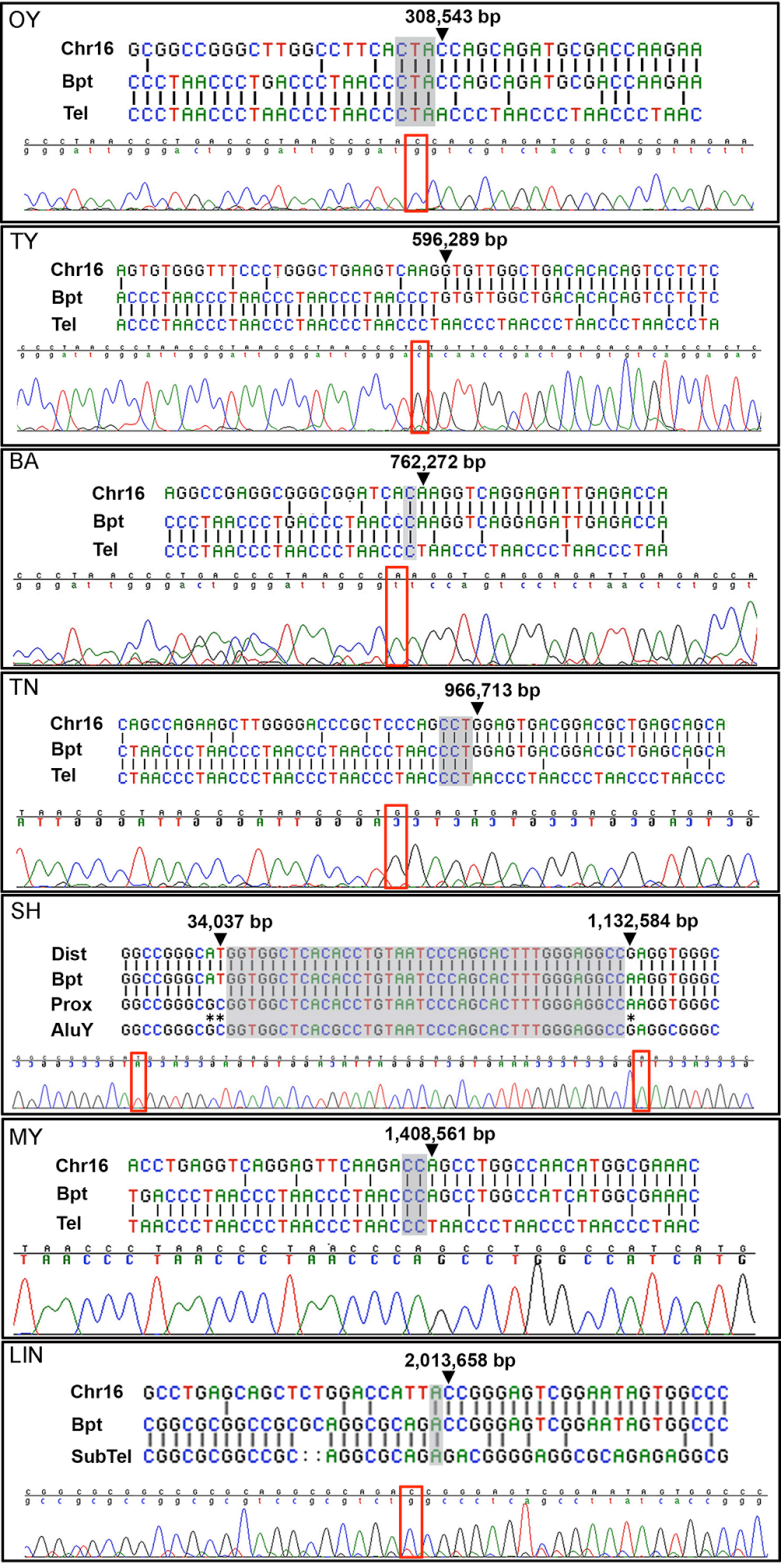
Chromosome 16 breakpoint sequences. DNA sequences at ATR-16 breakpoints. Patient codes are given in the upper left of each panel. For each case, alignment of the two normal sequences is shown with sequence from the derivative chromosome (upper) with chromatogram traces traversing each breakpoint (lower). Areas of ambiguity are highlighted with grey boxes and the location of the last unambiguous base pair(s) are denoted by arrowheads and red boxes. Chr16, normal chromosome 16 sequence; Bpt, breakpoint sequence; Tel, telomere repeat sequence; SubTel, subtelomere repeat sequence; Prox, proximal chromosome 16 sequence; Dist, distal chromosome 16 sequence; AluY, AluY repetitive element. Asterisks indicate informative polymorphisms allowing sequence origins to be identified. For patients MY and OY, a telomere primer with a mismatched G nucleotide was used.

**Figure 2:**
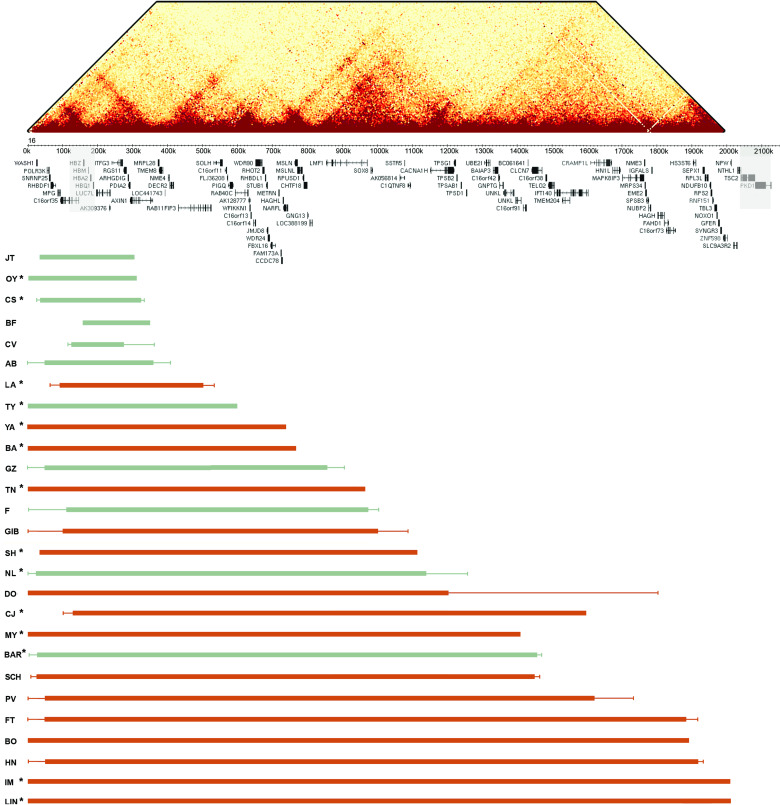
Summary of ATR-16 deletions. Upper: HiC interaction map showing interactions across the terminal 2 Mb of chromosome 16 at a 5 kb resolution in K562 cells (data from Rao *et al* 40). This shows how the ATR-16 deletions detailed in the lower section may impact the genome organisation. Middle: the positions of the α-globin cluster and other genes within this region are indicated. The α-globin genes and genes that, when mutated, are associated with tuberous sclerosis and adult polycystic kidney disease are shown in shaded boxes. Lower: the extent of each deletion is shown with the patient code (left). Deletions shown in green cause no other abnormalities apart from α-thalassaemia and those in red cause at least one other abnormality present in ATR-16. Solid bars indicate regions known to be deleted and fine lines show regions of uncertainty. Asterisks indicate individuals whose deletion breakpoints have been cloned or refined in this work.

In addition to the α-thalassaemia, manifesting as a microcytic anaemia (identified by full blood count) that is always present, common features of ATR-16 syndrome include speech delay, developmental delay and a variable degree of facial dysmorphism and, in severe cases, abnormalities of the axial skeleton. Individual case reports are provided in online supplementary information; newly cloned breakpoint sequences are shown in figure 1; deletions are shown in figure 2 and phenotypic abnormalities are summarised in table 1. Deletions larger than 2000 kb including the *PKD1* and *TSC2* genes lead to severe MR with polycystic kidney disease and tuberous sclerosis, respectively.^13^

**Table 1:** ATR-16 syndrome phenotypic severity

Case*	**Sex**	**Deletion coordinates (hg18**)	**Methods**	**Origin**	**Mechanism**	**MR**	**AT**	**SD**	**DD**	**FD**	**SA**	**Reference(s**)
JT	F	34,113 bp to 301,556 bp†	F, SB, S	Mat	De novo	–	+	–	–	–	–	Horsley, 2001^9^
OY‡	F	0 bp to 308,540 bp	F, SB, S	Pat	De novo	–	+	–	–	–	–	This study
CS‡(+1)§	F	~36,766 bp to 328,247 bp	A	Pat	Inherited	–	+	–	–	–	–	This study
BF (+5)§	M	166,680 bp to 342,681 bp	WGS	na	De novo	–	+	–	–	–	–	Heireman *etal* 24
CV	F	~1 22 000 bp to 2 99 000–3 75 000 bp	M	na	na	–	+	–	–	–	–	Coelho *etal* 41
AB	na	0–45,799 bp to 3 50 916–4 00 279 bp	M	na	na	–	+	–	–	–	–	Harteveld *etal* 42
LA‡	M	~94,214bp to 502,227 bp	A	Pat	Inherited	–	+	+	+/-	–	–	This study
TY(MI)‡	M	0 bp to 596,289 bp	F, SB, S	na	na	–	+	–	–	–	–	This study
TY(Mi)‡	F	0 bp to 596,289 bp	F, SB, S	Pat	Inherited	–	+	–	–	–	–	This study
YA‡	F	0 bp to 747,840 bp	F, A	na	na	na	+	+	+	+	–	This study
BA‡	F	0 bp to 762,370 bp	F, SB, S	Pat	De novo	–	+	–	+	–	–	Daniels *etal* 43
GZ	M	0–45,799 bp to 8 69 698–9 00 907 bp	M	Mat	Inherited		+	–	–	–	–	Harteveld *etal* 18
TN(Pa)‡	F	0 bp to 966,710 bp	F, SB, S	Mat	De novo	+/-	+	+	+	+	–	Daniels *etal* 43
TN(Pe)‡	M	0 bp to 966,710 bp	F, SB, S	Mat	Inherited	+	+	+	+	+	–	Daniels *etal* 43
TN(Al)‡	M	0 bp to 966,710 bp	F, SB, S	Mat	Inherited	+	+	+	+	+	–	Daniels *etal* 43
FI.2	F	0–45,799 bp to~9 76 591 bp	M	na	na	–	+	–	–	–	–	Bezerra *etal* 20
FII.1	M	0–45,799 bp to~9 76 591 bp	M	Mat	Inherited	–	+	–	–	–	–	Bezerra *etal* 20
FII.2	M	0–45,799 bp to~9 76 591 bp	M	Mat	Inherited	–	+	–	–	–	–	Bezerra *etal* 20
FII.4	F	0–45,799 bp to~9 76 591 bp	M	Mat	Inherited	–	+	–	–	–	–	Bezerra *etal* 20
FIII.1	M	0–45,799 bp to~9 76 591 bp	M	Mat	Inherited	–	+	–	–	–	–	Bezerra *etal* 20
GIB	F	~1 00 000 bp to~1,000,000 bp	F, A	na	De novo	+	+	+	+	+	–	Gibson, 2008^44^
SH(Pa)‡¶	M	34,037 bp to 1,132,584 bp	F, SB, S	Mat	Inherited	+	+	na	+	+	+	This study
SH(Ju)‡¶	F	34,037 bp to 1,132,584 bp	F, SB, S	na	na	–	+	na	–	–	–	This study
NL‡	M	0–23 949 bp to~1,246,849 bp	A, M	na	De novo	–	+	–	–	–	–	Phylipsen *etal* 45; This study
DO	F	0 bp to 1,175,000–1,805,487 bp	SB	Mat	Unknown	+	+	+	+	+	–	Wilkie *etal* 5
CJ‡	M	120,000 bp to 1,357,000 bp	F, A	Mat	De novo	+	+	+	+	+	+	This study
MY‡	F	0 bp to 1,408,950 bp	F, SB, S	Mat	De novo	+	+	+	+	+	–	This study
BAR‡	M	0–23,949 bp to~1,440,000 bp	A, M	na	De novo	–	+	–	–	–	–	This study
SCH	M	~281,65 bp to 1,447,989 bp	F, A, M	na	De novo	+	+	+	+	+	+	Scheps *etal* 28
PV	M	0–45,799 bp to 1,615,979–1,730,426 bp	M	na	De novo	+	+	+	+	+	+	Harteveld *etal* 18
FT	F	0–45,799 bp to 1,880,277–1,913,866 bp	M	na	De novo	+	+	+	+	+	+	Harteveld *etal* 18
BO	M	0 bp to 1,886,763 bp	C, F, SB, S	Pat	De novo	+	+	na	+	+	+	Wilkie *etal* 5; Lamb *etal* 46; Daniels *etal* 43
HN	M	0–45,799 bp to 1,913,923–1,928,982 bp	M	na	De novo	+	+	+	+	+	+	Harteveld *etal*, 2007^18^
IM‡	F	0 bp to 2,011,646 bp	F, SB, A	na	na	+	+	–	+	+/-	+	Felice 47; Fei *etal* 48; Daniels *etal* 43
LIN‡	F	0 bp to 2,013,657 bp	F, SB, S	Pat	De novo	+	+	+	+	+	–	Lindor *etal* 49; Daniels *etal* 43

+ indicates presence of an abnormality; – indicates absence and +/− indicates borderline assessment.

Methods column summarises the methods used to refine or identify the breakpoint:C, cytogenetics; F, FISH; WGS, Whole Genome Sequencing; M, MLPA; SB, Southern blot; A, microarray, S, breakpoint has been DNA sequenced.

*ATR-16 individuals are identified by unique codes, references are shown in figure 2. Pale green rows indicate ATR-16 individuals with only alpha-thalassemia, yellow rows indicate ATR-16 individuals also have at least one other abnormality but no defects of the axial skeleton and orange rows indicate the individual also has skeletal defects.

†40 bp ambiguity, values taken from midpoint

‡Indicates individuals whose deletion breakpoints have been cloned or refined in this work.

§There numbers refer to other family members who carry this deletion and have no associated abnormalities apart from alpha-thalassemia

¶Individuals have discordant abnormalities, most likely due to a deletion in *NRXN1*.

A, microarray; AT, alpha-thalassaemia; C, cytogenetics; DD, developmental delay; F, FISH; FD, facial dysmorphism; M, Multiplex Ligation-dependent Probe Amplification (MLPA); MR, mental retardation; na, data not available; S, breakpoint has been DNA sequenced; SA, skeletal abnormalities; SB, Southern blot; SD, speech delay; WGS, whole genome sequencing.

Twelve individuals from 9 pedigrees are reported here for the first time (OY, CS, CS (father), LA, TY (MI), TY (Mi), YA, SH (P), SH (Ju), CJ, MY and BAR) and we refine or identify the breakpoints in five previously reported cases (BA, TN, IM, NL, LIN). We define breakpoints at the DNA sequence level in 7 of the 14 pedigrees studied (figure 1), 6 of which have been repaired by the addition of a telomere or subtelomere. In the remaining family (SH), the deletion is interstitial and mediated by repeats termed short interspersed nuclear elements.

### Identification of co-inherited deleterious loci

Six individuals from four families (LA, BA, YA and TN) have 16p13.3 deletions smaller than 1 Mb and yet show relatively severe abnormalities. To test whether 16p13.3 deletions of <1 Mb may be unmasking deleterious mutations on the intact chromosome 16 allele in severely affected patients, we performed WGS where DNA was available (YA and the three affected members of the TN family) and considered only coding variants in the hemizygous region of chromosome 16. However, only common variants (allele frequency >5%) were present (online supplementary table 3) suggesting the cause(s) of the relatively severe phenotypes in these patients reside elsewhere in the genome. To identity rare variants genome-wide, we considered only those absent from the publicly available databases. This analysis yielded 14 variants shared between the three affected individuals of family TN (online supplementary table 4). Of these, only one (chr15:64 782 684 G>A) affects a gene possibly involved in the broader ATR-16 phenotypic abnormalities. This change leads to a R12X nonsense mutation in *SMAD6*, a negative regulator of the bone morphogenetic protein signalling pathway. Heterozygous mutations in *SMAD6* have been reported to underlie craniosynostosis, speech delay, global developmental delay, fine motor impairment and aortic valve abnormalities with variable penetrance (see ‘Discussion’ section). We checked inheritance of the *SMAD6* R12X variant in members of the TN family where samples were available and found it most likely came from the unaffected grandmother (individual I,2 in online supplementary figure 4). A phenotypically normal elder sister also inherited this variant. These findings suggest that coinheritance of this *SMAD6* loss of function variant with the chromosome 16 deletion may lead to the increased severity of the ATR-16 syndrome.

Further evidence that the effect of ATR-16 deletions is modified by other loci comes from patients SH (Ju) and SH (Pa), who harbour the same chromosome 16 deletion. Patient SH (Pa) has developmental delay and skeletal abnormalities, however, his mother SH (Ju) does not have craniofacial nor skeletal abnormalities nor developmental delay, although she suffers from severe anxiety and depression (see figures 1 and 2 and online supplementary information). Genome-wide microarray analysis revealed that both SH (Ju) and SH (Pa) harbour a ~133 kb deletion on the short arm of chromosome 2 including exons 5–13 of *NRXN1* (online supplementary figure 1).

### Chromatin structure

Recent reports demonstrate chromosomal rearrangements, including deletions, can result in aberrant DNA domain topology and illegitimate enhancer-promoter contact causing gene misexpression.^14^ Chromatin contact frequency is shown for the terminal 2 Mb of chromosome 16 in figure 2 to illustrate the effect of the deletions reported here on the chromatin structure. The deletion in BA removes ~50% of the self-interacting domain in which *CHTF18*, *RPUSD1*, *GNG13* and *LOC388199* reside, thereby potentially removing *cis*-acting regulatory elements of these genes, although the genes themselves remain intact. In the case of CJ, the deletion brings the powerful α-globin enhancer cluster^15^ into proximity of *CRAMP1L* and may cause its aberrant expression in developing erythroblasts. Although topologically associating domains have been reported to be stable structures,^14^ many chromatin contacts are now known to vary in a tissue-specific fashion^16^ and therefore it is not possible to predict which genes may be aberrantly expressed in any given tissue as a result of the ATR-16 deletions.

### Compensatory gene expression

**Figure 3:**
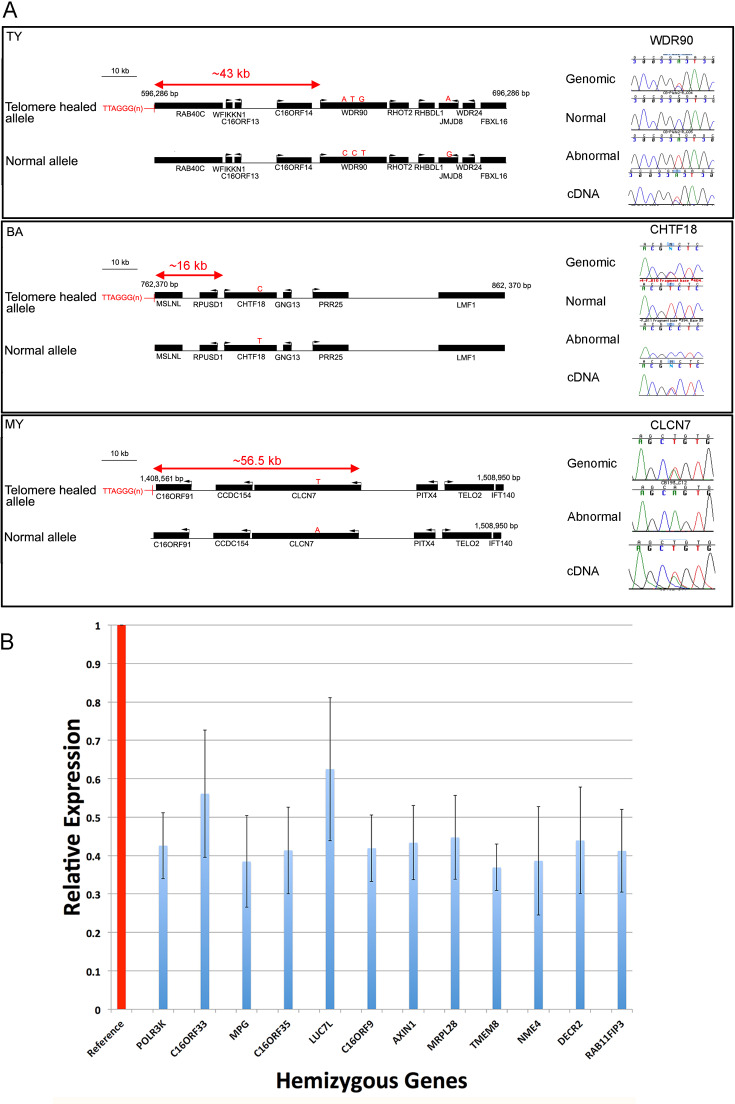
Effect of breakpoints and deletions on gene expression. (A) Schematic view of breakpoint positions in three patients with nearby expressed polymorphic genes. Genes are represented by black bars and transcription direction is indicated by an arrow. Polymorphic bases are shown by red letters indicating variant alleles and the distance of the promoter of each measured gene from the breakpoint is shown. On the right of each panel chromatograms show the quantity of the allele present in genomic DNA and cDNA from patient lymphoblastoid cells. (B) Expression of 12 genes within 500 kb of the tip of the short arm of chromosome 16 in lymphoblastoid cells from 20 normal individuals, shown as reference (red column) and from 11 ATR-16 individuals hemizygous for each gene. Measurements in control cells are normalised to 1 (red column), relative expression in ATR-16 patient cells is shown in blue. Error bars show SD. Gene expression was measured in triplicate and data combined.

One explanation for the relatively mild abnormalities in many cases of ATR-16 syndrome with deletions up to 900 kb may be compensatory transcriptional upregulation of the homologues of deleted genes on the undamaged chromosome 16. This has been described as part of the mechanism of genetic compensation, also termed genetic robustness.^17^ To assay for compensatory gene transcription, we used qPCR to measure expression of 12 genes within the terminal 500 kb of chromosome 16 in lymphoblastoid cells from 20 normal individuals and from 11 patients with monosomy for the short-arm of chromosome 16 and found no evidence of compensatory upregulation: transcripts of all deleted genes were present at ~50% of the normal levels in these cells (figure 3B). It is possible that other genes in downstream pathways affected by haploinsufficiency may be transcriptionally upregulated, however, the mechanisms underlying this are complex and beyond the scope of this study.

### Telomere position effect

To determine the effect of telomere proximity on genes adjacent to telomere-healed breakpoints, we measured their expression relative to the allele present in a normal chromosomal context. To achieve this, we screened them for informative SNPs in EBV transformed lymphoblastoid cells generated from ATR-16 patients. The phase of polymorphisms was established using MEL cells fused to patient cells and selected to contain a single copy of human chromosome 16, generated as previously described.^10^ Expressed coding polymorphisms were present in genes whose promoters are <60 kb away from breakpoints in three patients: TY, MY and BA.

For TY, the nearest gene expressed in lymphoblastoid cells containing a coding polymorphism is *WDR90*, the promoter of which is ~43.1 kb from the abnormally appended telomere (figure 3A). For BA, *CHTF18* is the closest expressed polymorphic gene with the promoter ~16.3 kb away from the breakpoint. For MY, *CLCN7* is the closest gene expressed in lymphoblastoid cells to contain a polymorphism, the promoter of this gene is ~56.1 kb away from the telomere stabilised lesion. To determine whether either allele of each of these three genes is silenced we prepared genomic DNA and cDNA from each cell sample and Sanger sequenced amplified fragments containing informative polymorphisms. We compared peak heights of polymorphic bases in chromatograms derived from cDNA and genomic DNA. None of the alleles assayed in the three patients tested showed any evidence of a repressive effect (figure 3A).

## Discussion

We characterised deletions leading to simple monosomy of the short arm of chromosome 16 that cause ATR-16 syndrome. Many ATR-16 patients suffer from neurodevelopmental abnormalities and one of the main questions in this disease, and in the study of CNVs in general, is how deletion size relates to phenotypic abnormalities. The monosomies analysed here show the likelihood and severity of neurological and developmental abnormalities increases with deletion size, however, there is no clear correlation.

The deletions in patients reported and reviewed here range from ~0.177 to ~2 Mb. Previous studies suggest the critical region leading to abnormalities in addition to α-thalassaemia is an 800 kb region between ~0.9 and~1.7 Mb from the telomere of chromosome 16 p^18^ and *SOX8* has been proposed as the critical haploinsufficient gene.^19^ However, a report of a family with no developmental delay nor MR harbouring a 0.976 Mb deletion, suggests deletions of *SOX8* may not lead to MR with complete penetrance and any ‘critical region’ for MR must start after this point^20^ (family ‘F’ in figure 2 and table 1). Supporting this we report patients NL and BAR, who have deletions of ~1.14 and ~1.44 Mb, respectively and show no abnormalities beyond α-thalassaemia.

By contrast, we find LA (deletion ~408 kb) has speech delay and YA (deletion ~748 kb) has speech and developmental delay and facial dysmorphism (figure 2, table 1). Family members of YA also have omphalocele, umbilical hernia and pyloric stenosis suggesting there are other loci rendering YA susceptible to developmental abnormalities. BA (deletion ~762 kb), who has a similarly sized deletion to YA, has developmental delay but no other abnormalities. Three other patients with deletions <1 Mb (TN (Pa), TN (Pe) and TN (Al)) have speech delay and facial dysmorphism. This suggests the risk of developmental and neurological abnormalities arises from much smaller terminal chromosome 16 deletions (~400 kb) than previously reported.

In family SH, we have identified a strong candidate for the discordant abnormalities: deletion of *NRXN1. NRXN1* encodes a cell surface receptor involved in the formation of synaptic contacts and has been implicated in autism spectrum disorder, facial dysmorphism, anxiety and depression, developmental delay and speech delay.^21^ There is a higher incidence of autism in males than in females, with a ratio of 3.5 or 4.0 to 1.^22^ This phenomenon is also specifically found in individuals with autism resulting from rearrangements of *NRXN1* 23, where two affected siblings inherited a deletion of *NRXN1* from their unaffected mother. It is therefore possible SH (Ju) is protected by her gender from the effects of *NRXN1* disruption while the neurological and skeletal abnormalities in SH (Pa) arise from the complex interaction of *NRXN1* perturbation with his gender and coinheritance of the 16p13.3 deletion. Abnormalities in siblings of YA and BF^24^ also suggest there may be other predisposing genes. Such loci compromise genetic robustness proposed to minimise the effect of deletions and loss of function mutations.^17^ Another example is the *SMAD6* R12X nonsense mutation present in all three affected members of family TN. Some patients with loss of function mutations in *SMAD6* have neurological abnormalities^25^ while others have not,^26^ suggesting variable penetrance. Our analysis shows there are no likely pathogenic variants on the hemizygous region of chromosome 16 in TN, suggesting modifying loci are present elsewhere in the genome. These may be rare variants (such as those identified in the TN and SH families) or common variation; a recent study shows that common genetic variants (allele frequency >5% in the general population) contribute 7.7% of the variance of risk to neurodevelopmental disorders,^27^ highlighting the complexity of this area.

Together these observations suggest that monosomy for 16p13.3 unmasks the effects of other variants genome-wide. This is supported by findings in SCH who has a very similar deletion to BAR and is more severely affected possibly owing to the presence of other CNVs.^28^ At the other end of the spectrum, large ATR-16 deletions may be associated with relatively mild abnormalities. In LIN (16p13.3 deletion ~2000 kb), there are no abnormalities of the axial skeleton and very mild facial dysmorphism. Similarly, in the case of IM (deletion size ~2000 kb), facial abnormalities are very mild and there is no evidence of language delay. Here, we propose chromosome 16p13.3 deletions larger than 400 kb predispose to MR and associated developmental abnormalities, however, we find no evidence for critical regions that incrementally worsen ATR-16 syndrome abnormalities.

In this work, we were able to provide evidence that CNVs and other variation genome-wide is likely to impact ATR-16 severity. However, we would not recommend this approach is yet widely applied as the impact of novel CNVs and sequence variants is challenging to interpret, especially when co-inherited with a terminal chromosome 16 deletion. We were unable to expand genome-wide analyses beyond the six patients (SH (Pa), SH (Ju), TN (Pa), TN (Pe), TN (Al) and YA) studied by microarray or WGS here and so cannot exclude the possibility variation genome-wide may influence the presentation of other ATR-16 patients reported and reviewed here. Previous work in human cells has shown that telomeres may affect chromatin interactions at distances of up to 10 Mb away from the chromosome ends^29^ reducing expression of the intervening genes. This phenomenon, termed TPE, is thought to be mediated by the spreading of telomeric heterochromatin to silence nearby genes. In budding yeast, this effect can extend a few kb towards the subtelomeres, although in some cases yeast telomeres can loop over longer distances^30^ and repress genes up to 20 kb away from the end of the chromosome. However, we could not detect compensatory upregulation of the homologues of deleted genes. Recently, a case of ATR-16 was reported with a~948 kb deletion who presented with a neuroblastoma in utero.^31^ These authors speculate that haploinsufficiency of the tumour suppressor *AXIN1* may have contributed to the neuroblastoma. Our finding that the remaining *AXIN1* allele shows no compensatory expression supports this hypothesis.

Terminal chromosome deletions are the most common subtelomeric abnormalities.^32^ The 16p deletions reported here are among the most common terminal deletions along with 1p36 deletion syndrome, 4p terminal deletion (causing Wolf-Hirschhorn syndrome), 5p terminal deletions (causing Cri-du-chat syndrome), 9q34 deletion syndrome and 22q terminal deletion syndrome. Despite their impact on human health, the mechanisms and timing underlying telomeric breakage remain unknown. Findings of terminal deletions of 16p reported and reviewed here and smaller deletions previously reported by our laboratory^33^ compared with more complex rearrangements at 1p, 22q and 9qter implies different chromosomes are predisposed to different breakage and rescue mechanisms. ATR-16 deletions are equally likely to have arisen on the maternal or paternal chromosome. There is no evidence that the parental origin affects the phenotypic severity of the ATR-16 syndrome, suggesting imprinting does not play a role in ATR-16 pathogenesis.

The presence of high and low copy number repeats at breakpoints may play a role in stimulating the formation of non-recurrent breakpoints.^34^ Low copy repeats (LCRs) are also mediators of non-allelic homologous recombination^35^ and could be involved in chromosome instability leading to terminal deletion. Following breakage, chromosomes can acquire a telomere by capture or de novo telomere addition, which is thought to be mediated by telomerase and this is stimulated by the presence of a telomeric repeat sequence to which the RNA subunit of telomerase can bind.^36^ We found 5 out of 6 telomere healed events share microhomology with appended telomeric sequence. This is the same ratio (5 out of 6 breakpoints with microhomology) described by Flint *et al* and supported by Lamb *et al* (1 out of 1) giving a total 11 out of 13 reported telomere-healed breaks characterised on 16p13.3 share microhomology with appended telomere sequences, strongly suggesting a role for internal telomerase binding sites.^37^ It may also be that telomerase binding to internal binding sites may inappropriately add telomeres and thereby contribute to the generation of the breakpoints.

The lack of evidence for TPE in silencing gene expression is surprising and at variance with previous findings,^38^ which show that TPE can influence gene expression at least 80 kb from the start of telomeric repeats. However, TPE is likely to be context and cell type dependent. Additionally, because of the lack of informative expressed polymorphisms in the patients studied here it was not possible for us to assay expression of genes immediately adjacent to telomeres and a more comprehensive screen may reveal TPE-mediated gene silencing closer to the telomere. Additionally, when the area of chromatin interaction (visualised by HiC) is considered (figure 2), contact domains for many genes adjacent to chromosomal breaks are severely disrupted. This is likely to include the loss of *cis*-acting regulatory elements and may bring the genes under the control of illegitimate regulatory elements.^39^ Therefore, it is likely that genes adjacent to breakpoints would be incorrectly spatiotemporally expressed.

This work substantially increases the number of fully characterised cases of ATR-16 syndrome reported and provides a uniquely well-characterised model for understanding how sporadic deletions giving rise to extended regions of monosomy may affect phenotype. The findings show larger deletions have a greater impact, but importantly our analysis suggesting there is no critical region defining the degree of phenotypic abnormalities has important implications for genetic counselling. Analysis of patients with uncomplicated deletions also revealed unexpected background genetic effects that alter phenotypic severity of CNVs.
