# Supplementary material for: ATR-16 syndrome: mechanisms linking monosomy to phenotype
Source: J Med Genet. 2020 Jan 31;57(6):414–21. doi: 10.1136/jmedgenet-2019-106528 (PMC7279195; doi:10.1136/jmedgenet-2019-106528)
Supplement: Supplementary data [file jmedgenet-2019-106528supp001.pdf]

Supplementary Information:

Case Reports

Female JT had microcytic anaemia but was otherwise normal. She was found to have an interstitial deletion of 267.4 kb that arose de novo on the maternal chromosome and the breakpoint was mapped by FISH and Southern blotting before being characterised at the sequence level by inverse PCR and found to have occurred within an Alu repetitive element (Horsley et al., 2001).

Female OY had microcytic anaemia but was otherwise normal. Results of fluorescence *in situ* hybridisation (FISH) show she has a deletion on the paternal chromosome 16 which arose *de novo* and that the breakpoint lies within cosmid 419C1 (Supplementary Table 1), revealing a truncation of ~308 kb. The breakpoint was refined by Southern blotting and identified by cloning and DNA sequencing of telomere-anchored amplification products. DNA sequence analysis shows telomeric repeats directly joined to the unique sequence (Figure 1). There is a 3 bp ambiguity, suggesting a minimal telomerase-binding site contributed to the stabilisation of this breakpoint by addition of a telomere. The last unique base is position 308,543 bp, interrupting the second intron of *AXIN1* (Figure 2). There are no repeats within 300 bp either side of the break.

Female patient CS was initially referred at the age of 2 months because of an abnormal head shape, which was shown by three dimensional computed tomography scan of the skull to be associated with bicoronal craniosynostosis. Screening known craniosynostosis-associated genes by DNA sequencing and for copy number variation by MLPA revealed no pathogenic variants. However, genome-wide microarray analysis (Agilent ISCA 60K oligoarray) revealed a ~290 kb deletion, affecting 15 coding genes, between 36,766 bp and 328,247 bp on chromosome 16, which was found to have been inherited from the patient's unaffected father. CS was reported to have normal developmental milestones at the age of 8 months and there were no concerns raised about her eating, sleeping or any aspect of her behaviour. She attended normal preschool from the age of four and on follow-up at the age of 5 years there were no reported concerns about her development. Although the craniosynostosis in this patient is likely to be genetic in origin, given the absence of this phenotype in all other cases with 16p13.3 deletions, including her transmitting father, it is unlikely to be related to her chromosome 16

deletion; neither the patient nor her father have any abnormalities indicative of broader ATR-16 syndrome.

Male BF was reported found to have α-thal trait and his deletion was identified by MLPA and characterised by low coverage WGS to be between 166,680 bp and 342,681 bp (Heireman et al., 2019). This patient was one of six confirmed deletion carriers in this family, one of whom (Patient 6) has symptoms consistent with ATR-16 syndrome, however, this did not segregate with the deletion and the authors conclude this to be unrelated to the chromosome 16 deletion as the remaining carriers exhibited only α-thalassemia.

Female CV (patient F in Coelho et al., 2010) presented with microcytic anaemia suspected of being α-thalassemia and was found by MLPA to have a deletion of ~177 kb including the entire α-globin locus and extending beyond the TMEM8A gene. This individual had no abnormalities beyond the α-thalassemia.

Individual AB was referred with a suspected haemoglobinopathy and was found by MLPA to harbour a terminal chromosome 16p deletion of with a maximum extent of 400 kb (Harteveld et al., 2005). This patient was not reported to have any abnormalities beyond α-thalassemia.

Male LA presented with α-thalassemia with language delay and hypotonia. However, he has no developmental delay or skeletal abnormalities. This patient also has a brother who harbours the same deletion and has significant developmental delay including speech delay, reduced attention span, behavioural problems and seizures. Microarray analysis revealed this patient to have an interstitial deletion of ~408 kb.

Individuals TY(MI) and TY(Mi) are father and daughter respectively and have no abnormalities apart from α-thalassemia. FISH suggests the 16p breakpoint lies within cosmid 338H10 (Supplementary Table 1), indicating a truncation of ~600 kb. The deletion was refined by Southern blotting and identified by Sanger sequencing of cloned telomere-anchored amplification products to be at 596,289 bp (Figure 1), in the second intron of *RAB40C* (Figure 2). The presence of telomeric repeats added directly to the breakpoint shows telomere mediated healing, however, there is no overlap at the breakpoint junction. The break occurs at a position 16 bp within the third iteration (of 6.5) of a 66 bp tandem repeat sequence. The repeat contains 2 ORFs, one similar to alpha collagen the other similar to AHA1, activator of heat shock 90kDa protein ATPase homolog 2 (yeast). These repeats are not present elsewhere in the genome. There is a long tandem repeat similar to MLT1E1 or

MLT1E2 located 221 bp centromeric of the breakpoint and 366 bp telomeric of the break there is an LTR variant (L1ME3A). It is possible that the presence of these repeated sequences contributed to the chromosomal leading to this deletion.

Female YA had speech delay (no words by the age of 2 years) and her development was delayed by 6-12 months. She has some facial dysmorphism with mild frontal bossing, prominent eyes, flat nasal bridge, low set ears and macroglossia. She also had short stature, inverted nipples, supernumerary nipples and a bifid uvula. YA showed evidence of microcytic anaemia and because of this she was referred to our laboratory for genetic testing. FISH studies showed a deletion at the tip of chromosome 16 with breakpoint at ~750 kb (between cosmid clones C444G9 and C335H7 – Supplementary Table 1). The maternal chromosomes appeared normal, however, as the father was lost to follow-up parental origin could not be established. High-resolution microarray refined the breakpoint to ~747,840 bp (Supplementary Figure 3) ~5 kb 5' of the *MSLN* gene in an area containing L1MB7 and L1MB8 LINE repeats and AluSx and AluSq/x SINE repeats that are likely to have contributed to the rearrangement.

Female patient BA was previously reported (Daniels et al., 2001) to have no physical abnormalities but does have speech delay and a lack of spatial awareness and visual memory. Cloning and DNA sequencing of telomere-anchored amplification products shows the breakpoint to be at 762,272 bp (Figure 1) disrupting the *MSLNL* gene by removing the last four exons. The break appears to be have been healed by telomerase as TAACCC repeats are immediately adjacent to the breakpoint and there is a potential 1 bp overlap. The break occurred at a position 65 bp into an AluY repeat, which may have contributed to an instability in this region.

Male patient GZ had surgery to repair pyloric stenosis shortly after birth and had persistent microcytic anaemia, however, there was no developmental delay nor any other abnormalities related to ATR-16 syndrome (Harteveld et al., 2007). MLPA revealed this patient to have a terminal deletion with a maximum extent of 900,907 bp with the breakpoint disrupting the *LMF1* gene.

Individuals TN(Pe) and TN(Al) are brothers and TN(Pa) is their mother. All three affected family members suffered from mild intellectual delay and developmental delay (6 months to 1 year delayed speech and walking). This family have been previously reported (Daniels et al., 2001), however, in this work we have identified the breakpoint using monochromosomal somatic cell hybrid mapping

followed by DNA sequencing of telomere anchored amplification products to reveal the lesion to be at 966,713 bp, (Figure 1). We also show that this deletion arose *de novo* on the maternal allele in Tn(Pa). The break interrupts the third exon of the *LMF1* gene (Figure 2) with no repeats in the vicinity. The break appears to have been healed by telomerase as there are TAACCC repeats joined with 3 bp of overlap suggesting a minimal telomerase recognition site which facilitated the telomere mediated healing (Figure 1). Whole genome sequencing of all three affected individuals revealed no rare coding polymorphisms on the extant chromosome 16 allele. However, all three affected individuals harboured a R12X variant in *SMAD6*, a gene which has previously been associated with developmental delay with variable penetrance.

The five individuals designated with the prefix F are from a Brazilian family reported in 2008 who were found to harbour a deletion of ~931 kb identified by MLPA (Bezerra et al., 2008). This deletion disrupted the *SOX8* gene and none of the family members showed any of the broader phenotypic abnormalities of ATR-16 syndrome. This was the first reported case that showed haplo-insufficiency for *SOX8* in humans doesn't cause phenotypic abnormalities.

Female patient GIB was initially presented with a mild hypochromic microcytic anaemia and the presence of HbH inclusions (Gibson et al., 2008). Developmental delay was diagnosed at the age of 2 years, however, she did not have any dysmorphic features at this stage. By the age of 6 years craniofacial abnormalities were reported including a high forehead, flattening of the maxilla and a high nasal root and bridge together with up-slanting palpebral fissures. Intellectual abilities were reported as being around the 4th centile. Together these features are all typical of the broader ATR-16 phenotype. Microarray and FISH analyses showed a *de novo* interstitial deletion of ~900 kb in the subtelomeric region of chromosome 16p13.3 likely to be responsible for causing the abnormalities present in patient Gib.

Patient SH(Pa) exhibited motor delay throughout childhood, he had an IQ of 48 when tested and has facial dysmorphism including micrognathia, maxillary hypoplasia and a high, arched palate. He also has skeletal abnormalities including clinodactyly, tapering fingers with narrow nails, a short 4th metacarpal, intoed gait, pes planus and genu valgus. Other abnormalities include crypto-orchidism and a hypoplastic scrotum. Monosomy for 16p13.3 was identified by FISH, with the break being found to be between clones C349E11 and C344F5, (Supplementary Table 1) and subsequently refined by Southern blotting and inverse PCR to obtain the

breakpoint sequence. This revealed the rearrangement to be an interstitial AluY mediated 1,098,548 bp deletion (between ~34037 bp to ~1132584 bp), the breakpoints of which do not directly disrupt a gene (Figure 2). Because this deletion is mediated by Alu repeats there is 38 bp of overlapping sequence, making it is impossible to define the precise breakpoint (Figure 1). However, minor variations in the repeat sequence allow the last unique bases on either side of the deletion to be determined (asterisks in Figure 1). The telomeric break removes the most distal gene on 16p13.3 (*POLR3K*) and the proximal break occurs between the *C1QTNF8* and *CACNA1H* genes (Figure 3). Interestingly this breakpoint appears to be mediated by the same AluY element associated with the interstitial deletion in JT (see Figure 2) suggesting this locus may be particularly prone to rearrangement.

Male NL was diagnosed with α-thalassemia at the age of 9 years, there were no signs of mental retardation, he followed a normal school education and had normal developmental milestones. At present he is 19 years of age and studies law and plays soccer in his local team. MLPA and subsequently microarray analysis (Phylipsen et al., 2012) showed this patient to have a distal deletion of ~1.14 Mb with the breakpoint falling between *C1QTNF8* and *CACNA1H* (Figure 2). This deletion is slightly larger than that identified in family SH, surprisingly, however, it is not associated with any abnormalities apart from α-thalassemia.

A large terminal deletion of at least 1 Mb was identified in case DO by Wilkie and colleagues (Wilkie et al, 1990) using Southern blotting. This patient has craniofacial abnormalities including hypertelorism, a prominent nasal bridge, a high palate with crowded upper teeth and a small chin. Skeletal examination was normal (apart from short stature) but she had a marked speech delay, having a very limited vocabulary at the age of five years.

Male patient CJ had a birth weight of 3.2 kg and was initially referred for genetic testing because of developmental delay, speech delay and slow cognitive, social and motor development at 14 months of age. He exhibited a range of dysmorphic features including a high, broad forehead, a long and flat midface, a high narrow palette, retrognathia, micrognathia and downturned palpebral fissures. Skeletal abnormalities included bilateral hallux valgus and bilateral clinodactyly of the 5th finger. He had a systolic ejection heart murmur. Because CJ was found to have microcytic anaemia in combination with these abnormalities a possible diagnosis of ATR-16 was pursued. FISH studies revealed an interstitial deletion of the terminal region of chromosome 16 shown by absence of probes intervening the

telomeric cosmid CRA36 and proximal cosmid C399E4 (Supplementary Table 1). The deletion in this patient was refined by high-resolution microarray, which revealed it to be between ~120,000 bp and ~1,357,000 bp on chromosome 16 (Supplementary Figure 3) giving a total deletion size of ~1.15 Mb (Figure 2).

Female patient MY had a birth weight of 2.7 kg, she initially fed poorly although her health improved following surgical repair of an atrial septal defect. MY's heart defect is unlikely to be linked to the ATR-16 syndrome as her sister also had an atrial septal defect yet did not inherit monosomy for chromosome 16p13.3, although this cannot be excluded. MY also has plageocephaly and mild telecanthus and hypertelorism. She had delayed speech and mild delay to her physical development (the patient walked at 18 months of age). Results of FISH with 16p cosmids suggest the breakpoint lies between cosmids 399E4 and 312E8 (Supplementary Table 1), indicating terminal deletion of approximately 1.4 Mb. Southern blotting followed by cloning and DNA sequencing of telomere-anchored amplification products showed the break occurred at 1,408,561 bp (Figure 1), between two tandem AluSx SINE repeats, flanked by the *C16orf91* and *UNKL* genes (Figure 2). There is a rearrangement at the breakpoint with complex deletion of part of the AluSx repeat (Supplementary Figure 2). Telomeric sequence is joined directly to the breakpoint with a 2 bp overlap, suggesting telomerase mediated healing (Figure 1).

Female BAR was diagnosed with α-thalassemia by the identification of HbH inclusions at the age of 7 years. She was found to have inherited the –α3.7 deletion from her mother on one allele and MLPA and subsequently microarray analysis revealed an interstitial deletion from ~0-23,949 bp to ~1,461,124 bp had arisen *de novo* on the other allele (Figure2 and Supp. Fig 3). BAR had no phenotypic abnormalities beyond her α-thalassemia.

Male patient SCH was referred with at the age of 8 years with HbH disease to a normal mother and a father a haematological profile with a+ -thalassemia (Scheps et al., 2016). In his neonatal period, he required hospitalisation for respiratory distress and hypotonia. He also showed developmental delay and other dysmorphic craniofacial features and abnormalities of the hands and lower limbs (Scheps et al., 2016). FISH, SNP-array and MLPA revealed this patient to have an interstitial deletion of ~1.2 Mb.

Male patient PV had developmental delay at the age of 2, at the age of 11 he was noted to have mildy dysnmorphic craniofacial features, his truck showed pectus

excavatum and hyperlaxity of his joints was observed (Harteveld et al., 2007). MLPA revealed this patient to have a terminal deletion with a maximum extent of ~1.7 Mb (Harteveld et al., 2007).

Female patient FT was referred at the age of 30 years because of mild microcytic hypochromic anaemia. In infancy it was noted she had a developmental delay (Harteveld et al., 2007). At the age of 31 examination revealed craniofacial abnormalities and abnormalities of the hands and feet including clinodactly and lateral deviation. MLPA revealed a large deletion of 16p13.3 with a maximum extent of ~1.9 Mb.

Male patient BO had a small head, mild ptosis, a small mouth and a long filtrum and bilateral talipes equinovarus (reviewed in Wilkie et al., 1990). The breakpoint was refined by Wilkie and colleagues and subsequently identified as a atr terminal deletion of ~1.9 Mb (Lamb et al., 1993).

Male patient HN was reported by Harteveld and colleagues (Harteveld et al., 2007). At the age of 10 months he had delayed motor development and hypotonia. Speech delay was reported at the age of 30 months and at 3 years he was found to have an active language delay. He also had typical facial features associated with ATR-16 including downslanting palpebral fissures, mild hyperterlorism, abnormal ears. His skeletal defects were mild with only a club foot (left) and a flat foot (right) reported). MLPA revealed this patient to have a large deletion of a 1.9 Mb.

The phenotypic abnormalities of female patient IM have previously been reported (Daniels et al., 2001; Fei et al., 1992; Felice et al., 1984). Briefly, she was found to have microcytic anaemic at the age of 5 months, her growth was slow and motor retardation was noted by the age of 1 year. IM was later reported to have to suffer developmental delay (behaving like a 5 year old at the age of 8), however, her language and adaptive development were considered normal. IM has mild facial dysmorphism (tall forehead and macrodontia) and bilateral clubbed feet that were surgically corrected in early childhood. Previous studies performed Southern blotting (Fei et al., 1992) and FISH (Daniels et al., 2001) to refine the deletion, here we conduct high-resolution microarrays that reveal a large terminal deletion of chromosome 16 with the breakpoint at ~2,011,646 bp. The relatively mild phenotypic abnormalities seen in this patient are surprising given the relatively large deletion of chromosome 16 identified.

Female patient LIN had expressive speech delay and developmental delay that has been described previously (Lindor et al., 1997). She has a degree of facial

dysmorphism including a tall, broad forehead and a broad nasal root and midface retrusion. Previous FISH studies identified a terminal deletion of chromosome 16 with the breakpoint ~ 2 Mb from the telomere (Daniels et al., 2001). In this study we further refined the deletion using Southern blotting and identified the junction fragment by DNA sequencing of telomere anchored amplification products to be at 2,013,658 bp (Figure 1). The breakpoint is flanked by the genes *NPW* and *SLC9A3R2* (Figure 2). There is a short region of subtelomeric sequence of unspecified chromosomal origin between the break and the telomeric repeats suggesting a recombination-based mechanism may have stabilised the chromosomal breakage in this case (Figure 1). The lack of skeletal abnormalities seen in LIN is surprising given that the deletion is over 2 Mb.

Supplementary Methods

Genes and Assay Numbers Used in Quantification of Gene Expression

POLR3K (Hs00363121_m1), *C16orf33* (Hs00430677_m1), *C16orf35* custom assay (Forward; CAACGCCCTCAGCTTTGG, Reverse; GCTGGTGAGGGTCATGTCATC, Probe; CCCCAACCAGCAGC), *LUC7L* (Hs00216077_m1), *AXIN1* (Hs00394718_m1), *MRPL28* (Hs00371771_m1), *TMEM8* (Hs00430491_m1), *NME4* (Hs00359037_m1), *DECR2* (Hs00430406_m1), *Rab11FIP3* (Hs00608512_m1) *ACTB* (Hs99999903_m1). Two custom assays were obtained from Eurogentec (www.uk.eurogentec.com): *MPG* (Forward; 5'-GCATCTATTTCTCAAGCCCAAAG-3', Reverse; 5'-GGAGTTCTGTGCCATTAGGAAGTC-3', Probe; 5'-AGTTCTTCGACCAGCCGGCAGTCC-3') and C16orf9 (Forward; 5'-GGCGGCCCGTTCAAG-3', Reverse; 5'-GAGCCCACAAGAAGCACA-3', Probe; 5'-TCCCAGGGAACGCCGGTG-3'). Analysis was performed using the comparative C_T Method ($\Delta\Delta C_T$) (Livak et al., 2001). Data in Figure 3A were obtained by amplification and Sanger sequencing of informative polymorphisms from genomic DNA and cDNA.

Microarray Analysis

DNA from patient LA was tested using an 8 x 60K SurePrint G3 custom CGH + SNP microarray (Agilent) and analysed using Agilent Cytogenomics software 4.0. Genomic DNA from patients NL and BAR was analysed using a custom fine tiling array covering the alpha- and beta-globin gene clusters and surrounding areas was used (Roche NimbleGen, Madison, WI, USA). Array design was based on NCBI Build 36.1 (hg18) and used as previously described (Phylipsen et al., 2012). Genomic DNA from patients SH(Ju) and SH(Pa) was tested using CytoScan HD arrays (Affymetrix) and analysed using Karyoview software. Microarray analysis was performed with genomic DNA from patients CJ, IM and YA using the Sentrix Human CNV370 BeadChip (Illumina) and analysed using GenomeStudio software. DNA from patient CS was analysed using the Agilent ISCA (International Standards for Cytogenomic Arrays) 60K oligoarray.

Supplementary Figures

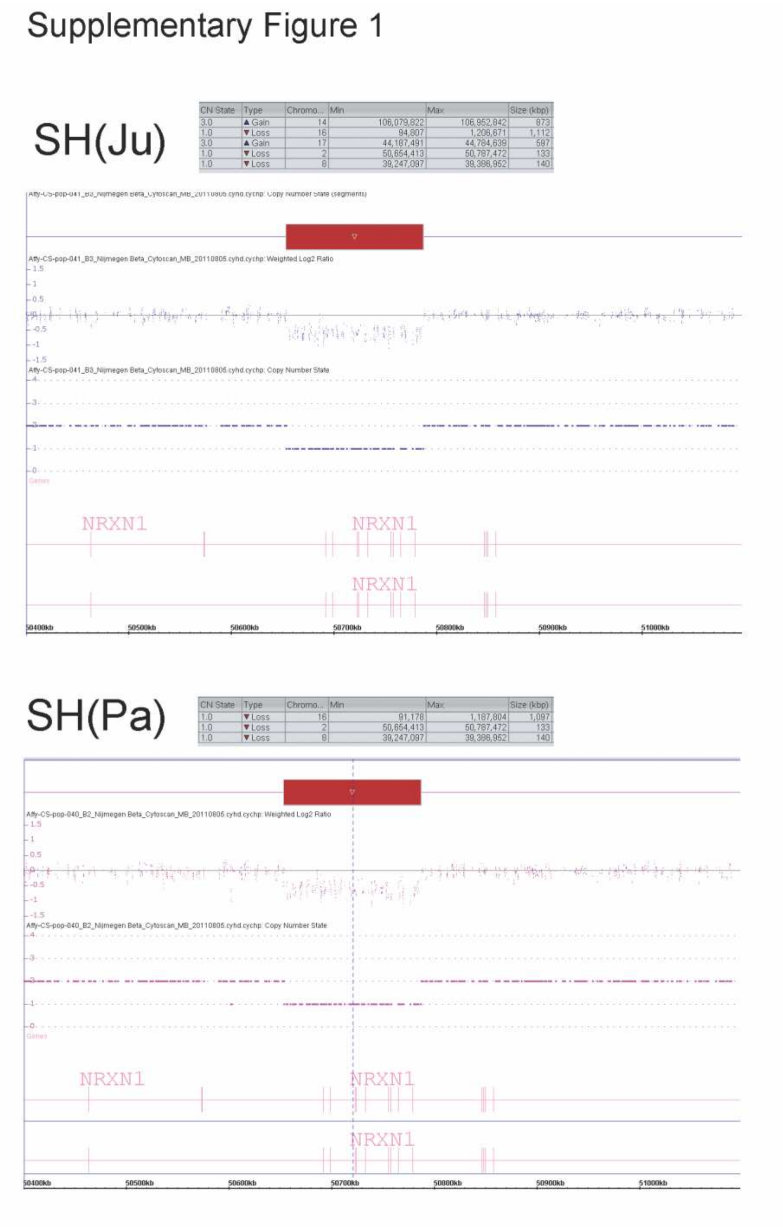

Supplementary Figure 1: Copy number variations (CNVs) identified in SH(Ju) and SH(Pa). Tables show CNVs called by Affymetrix Cytoscan software and figures below show the log2 ratio of probes and the copy number state for the chromosome 2 deletion encompassing exons 5 to 13 of *NRXN1*. Both individuals also harbour deletions of chromosomes 8 and 16. The chromosome 8 deletion disrupts the pseudogene *ADAM5* and *tMDC* encoding uncharacterised protein ENSP00000328747. The chromosome 16 deletion underlies the ATR-16 syndrome in these patients.

Supplementary Figure 2

MY breakpoint Seq:

```
AGCCTGGCCA tCATGGCGAA ACCtcacatg gtagcgggca ccaGTAATCC  50
CAGCTACTCa GGAGGCTGAG GCAGAAGAAt TGCTTGAACC CAGGAGACAA  100
GAGGTTGCAG TGAGCCAAAA TTGTGCCACT GCACTCCAGT CTGGGAGACA  150
GAGTGAGACC CTGTCTCAAA AATAAAAACA AAACAAAACA AAACAAAAAA  200
ACAAAAAACA ATCACTTAGG TAACCCAAAA TAGTGCCCAT CATTTGTGAA  250
AAGAAATACT CACTAGTatc ttgctgaaaa actcgagcca tccggaagat  300
ctggcggccg ctctccattt nagtgagtcg a
```

Genomic Chr 16:

```
gtatgaaaac aattgaccgg gcgcagtggc tcatgcctat aatcccagaa  1408510
ctttgggagg ccaaggcggg cagatcactt gaggtcagga atctgagacc  1408560
AGCCTGGCCA aCATGGCGAA ACCccgtctc tactaaaaat acaaaaatta  1408610
gacgggtgtg gcggtgcgtg cctGTAATCC CAGctaccct ggaggctgag  1408660
gcaggagagt cgcttgaacc caggaggtgg aggttgcagt gagccaagat  1408710
ggagccactg tactctagcc taggtgacag agcaagactc aatctcaaaa  1408760
aaaaaaagag aaaacaatct ggggccgggc gcggtggctc ccatctgtaa  1408810
tcccagcact gtgagaggcc gaggaggaga gatcacttga agtcaggagt  1408860
ttgagaccag cctggccaac atgccaaaag cccgtctcta ctgaaaattt  1408910
aaaaattatc cgggtgtggt ggcacaagcc tgtagttcta gCTACTCgGG  1408960
AGGCTGAGGC AGAAGAAcTG CTTGAACCCA GGAGACAAGA GGTTGCAGTG  1409010
AGCCAAAATT GTGCCACTGC ACTCCAGTCT GGGAGACAGA GTGAGACCCT  1409060
GTCTCAAAAA TAAAAACAAA ACAAAACAAA ACAAAAAAAC AAAAAACAAT  1409110
CACTTAGGTA ACCCAAAATA GTGCCCATCA TTTGTGAAAA GAAATACTCA  1409160
CTAGTtgtat tctactcaga aaatggttgc cttgcttaaa aagttttgca  1409210
gaaaactgca aaagttcctc atttctggta ctattcggta atatatatgt  1409260
aggtt
```

Side by Side alignment:

```
0000001 agcctggccatcatggcgaaacc 0000023
>>>>>>> |||||||||| |||||||||||| >>>>>>>
1408561 agcctggccaacatggcgaaacc 1408583
```

```
0000044 gtaatcccag 0000053
>>>>>>> |||||||||| >>>>>>>
1408634 gtaatcccag 1408643
```

```
0000054 ctactcaggaggctgaggcagaagaattgcttgaacccaggagacaagag 0000103
>>>>>>> |||||| ||||||||||||||||||| ||||||||||||||||||||||| >>>>>>>
1408952 ctactcgggaggctgaggcagaagaactgcttgaacccaggagacaagag 1409001

0000104 gttgcagtgagccaaaattgtgccactgcactccagtctgggagacagag 0000153
>>>>>>> |||||||||||||||||||||||||||||||||||||||||||||||||| >>>>>>>
1409002 gttgcagtgagccaaaattgtgccactgcactccagtctgggagacagag 1409051

0000154 tgagaccctgtctcaaaaataaaaacaaaacaaaacaaaacaaaaaaaca 0000203
>>>>>>> |||||||||||||||||||||||||||||||||||||||||||||||||| >>>>>>>
1409052 tgagaccctgtctcaaaaataaaaacaaaacaaaacaaaacaaaaaaaca 1409101

0000204 aaaaacaatcacttaggtaacccaaaatagtgcccatcatttgtgaaaag 0000253
>>>>>>> |||||||||||||||||||||||||||||||||||||||||||||||||| >>>>>>>
1409102 aaaaacaatcacttaggtaacccaaaatagtgcccatcatttgtgaaaag 1409151

0000254 aaatactcactagt 0000267
>>>>>>> |||||||||||||| >>>>>>>
1409152 aaatactcactagt 1409165
```

Supplementary Figure 2: DNA sequence of breakpoint in patient MY (upper) shown in comparison to genomic sequence with MY breakpoint sequence highlighted in blue (middle) and the side by side alignment of these sequences (lower). The breakpoint present in MY is complex and there is rearrangement of the Alu repeat associated with breakpoint.

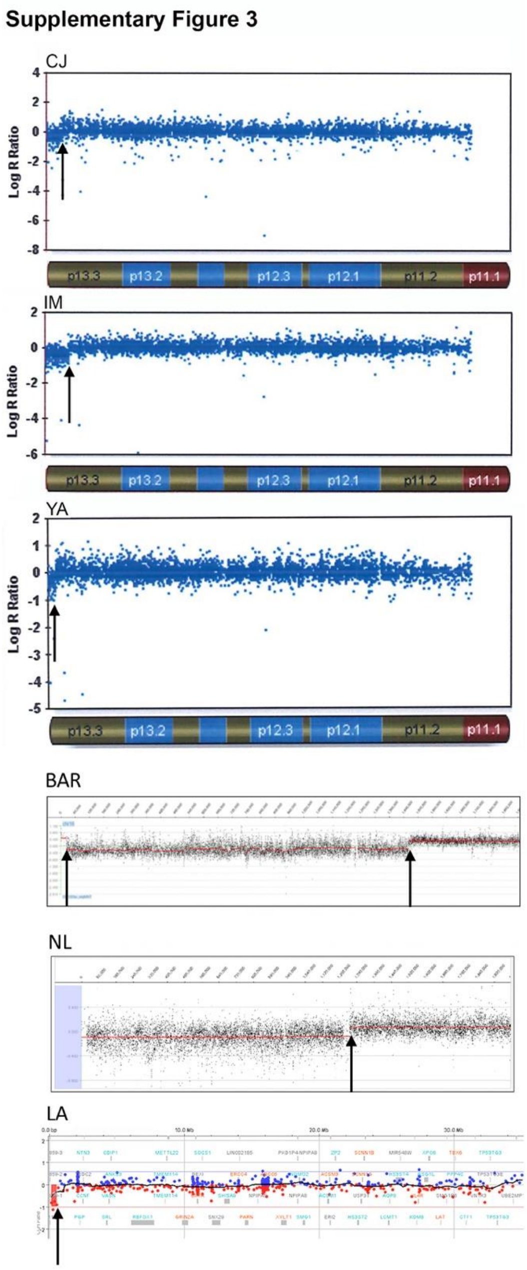

Supplementary Figure 3: Microarray data from patients CJ, IM, YA, BAR, NL and LA showing the LogR ratios of individual probes, arrows indicate loci at which analysis software identified the breakpoints.

Supplementary Figure 4

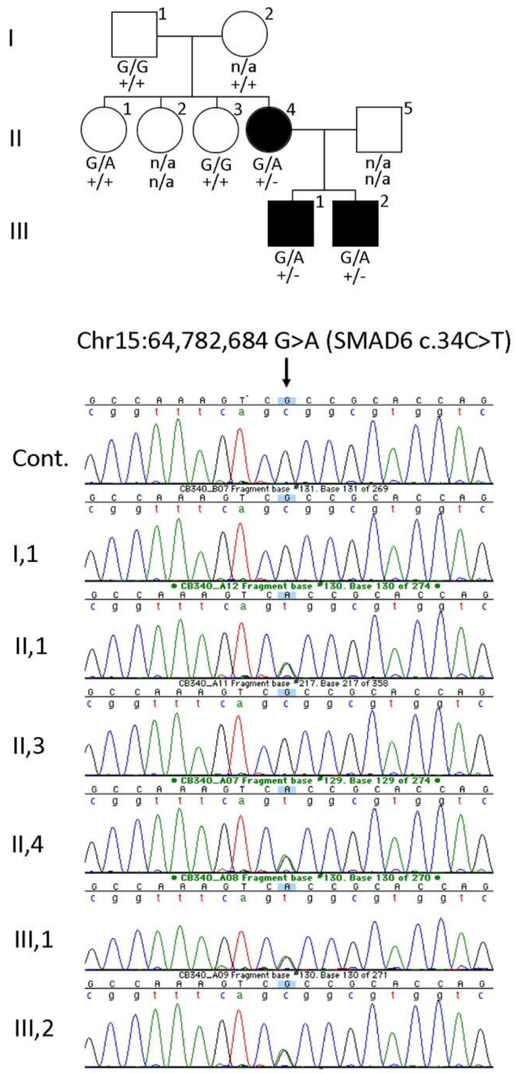

Supplementary Figure 4. Identification of SMAD6 change in Family TN. Affected family members are shown with filled symbols and unaffected individuals are represented with open symbols. Below each symbol the genotype at chr15:64,782,684 is written on the upper line, G is reference and A is the variant leading to the R12X nonsense change in *SMAD6*. The lower line of text indicates the ATR-16 deletion in this family, + indicates an intact chromosome 16 and – indicates the deleted allele. Because two siblings carry the *SMAD6* variant this is likely to have been inherited from the grandmother (I,2). Coinheritance of these variants may have led to the relatively severe ATR-16 abnormalities present in this

family. The lower part of the figure shows chromatograms for a control individual (Cont.) and family members as indicated.

Supplementary Table 1 FISH Probes

Probe	Coordinates	OY	TY	SH	MY	LIN	YA	CJ
Telomere Assay	na	N	nd	nd	N	nd	N	N
**16p cosmid**								
CRA36	52706-99963 bp	-						+
HSGG4	97541-111766 bp		-		-		-	
HSGG1	129137-172194 bp	-	-		-		-	-
Cos12	158777-203194 bp		-					-
C314G4	243192-285276 bp	-						
C419C1	265315-316860 bp	+/-	-					-
C338B10	297847-340834 bp	+						
C415C1	332201-381990 bp	+			-			
C356B8	485020-530746 bp	+						
C366D1	521427-565641 bp		-					
C407A10	557634-598294 bp		-					
C338H10	580029-610790 bp		+/-		-			
C398G5	594663-640361 bp	+	+					
C444G9	708336-751169 bp						-	
C335H7	737067-783265 bp						+	
C360B4	851308-895707 bp				-			
C313F9	1025418-1072203 bp	+						
C349E11	1068736-1111710 bp			-	-			-
C344F5	1122769-1160074 bp			+				
C320E12	1172104-1208298 bp							-
C399E4	1359308-1404844 bp				-			+
C312E8	1395863-1429931 bp				+			
C305C8	1461998-1505438 bp	+			+			+
C371H6	1755695-1799062 bp				+			
C439A6	1882600-1923854 bp	+						
910O19	1965888-2004708 bp					+/-		
1308N18	1996694-2036573 bp					+		
2171017	2038309-2078584 bp					+		

Presence of signals on both chromosome 16 homologues is indicated by "+", "-" indicates signal absent on one chromosome 16 homologue, "+/-" indicates weaker signal on one homologue compared to the other. The top row indicates patients who were analysed for subtelomeric rearrangements using a telomere assay (Knight et al., 1997 and Horsley et al., 2001): N, no rearrangement; nd, not done; na, not applicable.

Supplementary Table 2 Oligonucleotides used to clone breakpoints

Patient	Forward primer	Reverse primer
SH	5'-AGATACATGCTCCCAGTCTCA-3'	5'-CGTATATCTGGTCTCTATCTTC-3'
OY	5'-CAAAGCACGCATCCATAGGC-3'	5'-CCCTAACCCTGACCCTAACCC-3'
LN	5'- GCAGAGGGAGAGCAGGTCTCAG-3'	5'-CCCTAACCCTGACCCTAACCC-3'
MY	5'- CTGAAGGACTTGGCTGGTGGAT-3'	5'-CCTAACCCTGACCCTAACCC-3'
BA	5'-GAGCAAAGTACACAAACTGGGTGAC-3'	5'-CCTAACCCTGACCCTAACCC-3'
TN	5'-AACTGGCCTTGTCTGTGCCTTAAGCT-3'	5'-CCCTAACCCTGACCCTAACCC-3'
TY	5'-CCTACCACCAGCAAGAACGGA-3'	5'-CCTAACCCTGACCCTAACCC-3'

Supplementary Table 3 Deleterious Chromosome 16 Variants

Patients*	Gene#	Variant%	Identifier^	Freq$	ANNOVAR@
TN(Al, Pa, Pe)	MRPL28	H27Y	rs3194151	11%	3/6
TN (Al,Pe)	PICQ	G523S	rs7187227	14%	3/6
TN(Al) YA	PICQ	T14A	rs2071979	~49%	2/6
TN(Al, Pa,Pe) YA	POLR3K	S24A	rs3194151	100%	2/6
TN(Pa)	PDIA	T286M	rs2685127	8%	3/6
TN(Pa)	CHTF	S63F	rs2277902	5%	1/6
(Pa)	PRR25	T92S	rs1005190	41%	1/6
YA	NPRL3	R158fs	rs35963490	33%	6/6
YA	RGS11	G499A	rs9806942	15%	3/6
YA	RHOT	R425C	rs3177338	33%	3/6
YA	RHOT	R245Q	rs1139897	33%	2/6
YA	RGS11	T728C	rs739999	31%	1/6
YA	WDR90	H899Q	rs45613635	34%	1/6

*Codes for patients harbouring each of the changes listed are shown in this column. #Gene symbols are used. % Effects of variants on coding sequence. ^The unique identifier from dbSNP is listed for each variant. $ Allele frequencies as a population average, data from dbSNP. @ The pathogenicity of each variant was given a custom deleterious score based on a six-point scale, (Fu et al., 2013) calculated using output from ANNOVAR (Wang et al., 2010).

Supplementary Table 4. Shared novel variants in 3 affected members of family TN

Chr#	Position (bp)	Gene	Variant$	ANNOVAR%	Associated abnormalities^
1	1336598	PRAMEF27	Q144R	1/6	na
1	26671545	CRYBG2	L524fs	6/6	na
2	71190373	ATP6V1B1	R331W	4/6	Renal tubular acidosis with progressive nerve deafness.
3	141163642	ZBTB38	K804N	5/6	Potential role in human height variation (nearby GWAS association).
4	17061677	CLCN3	A290S	4/6	Loss of hippocampus in mouse KO.
5	179068881	C5orf60	splicing	6/6	na
8	125052168	FER1L6	G837D	3/6	na
11	1018248	MUC6	T1518I	2/6	Gastric cancer
11	62293804	AHNAK	F2695L	2/6	Neuroblastoma
13	103718412	SLC10A2	K63fs	6/6	Chrohn's disease, bile malabsorbtion.
14	33014538	AKAP6	L227V	5/6	na
15	66995630	SMAD6	R12X	6/6	Aortic Valve Disease, Craniosynostosis, developmental delay.
19	59010556	SLC27A5	V483M	6/6	na
20	5170758	CDS2	P406A	4/6	na

#Chromosome. $The effect of each variant on the protein is shown. %The custom pathogenicity score attributed to each variant (see Supp Table 3 notes). ^Annotation present in the OMIM database associated with each gene, na indicates no information in OMIM. The pathogenicity of each variant was given a custom deleterious score based on a six-point scale, (Fu et al., 2013) calculated using output from ANNOVAR (Wang et al., 2010).
